# Simple and Effective Squash-PCR for Rapid Genotyping of Industrial Microalgae

**DOI:** 10.3390/life14010115

**Published:** 2024-01-12

**Authors:** Guoliang Yuan, Song Gao, Jeffrey J. Czajka, Ziyu Dai, Kyle R. Pomraning, Rylan D. Duong, Beth A. Hofstad, Shuang Deng

**Affiliations:** 1 Chemical and Biological Processes Development Group, Pacific Northwest National Laboratory, Richland, WA 99352, USA; guoliang.yuan@pnnl.gov (G.Y.);; 2 Marine and Coastal Research Laboratory, Pacific Northwest National Laboratory, Sequim, WA 98382, USA

**Keywords:** Squash-PCR, genotyping, microalgae, colony PCR, genetic screening

## Abstract

Microalgae are recognized for their versatility in providing renewable energy, biopharmaceuticals, and nutraceuticals, attributed to their sustainable, renewable, and cost-effective nature. Genetic engineering has proven highly effective in enhancing microalgae production. PCR-based genotyping is the primary method for screening genetically transformed microalgae cells. Recently, we developed a novel PCR method, namely Squash-PCR, and employed it for the molecular analysis of industrially important fungi and yeasts. In this study, we successfully implemented the Squash-PCR technique in 12 industrially significant algae species. This approach offers a quick and reliable means of obtaining DNA templates directly from squashed algal cells, eliminating the need for time-consuming and labor-intensive cultivation and genomic DNA extraction steps. Our results demonstrate the effectiveness of Squash-PCR in detecting and characterizing target genes of interest in 12 different algae species. Overall, this study establishes the Squash-PCR method as a valuable tool for molecular studies in algae, enabling researchers to rapidly screen and manipulate genetic traits in diverse algal species.

## 1. Introduction

Microalgae have gained substantial interest from industrial sectors due to their capability to synthesize diverse biologically active compounds, exhibit rapid biomass growth, and adjust their biochemical composition in response to cultivation conditions [1]. These characteristics make microalgae an exceptionally versatile resource with applications spanning numerous sectors. As an example, microalgae have played multiple roles in pharmaceuticals, food production, wastewater treatment, and biofuel production [2,3]. Thus, these multifaceted microorganisms hold a pivotal position in advancing diverse industries, contributing to the development of sustainable and environmentally friendly technologies. Genetic engineering plays a crucial role in enhancing the traits of these microorganisms, offering opportunities for improved productivity and resource utilization [2]. Genotyping PCR techniques are particularly invaluable tools that enable rapid and precise screening of mutants to select strains with desired characteristics.

Colony PCR with microalgae is challenging due to their complex cell wall structure, which can hinder efficient genetic material extraction and results in a more labor-intensive and time-consuming PCR process. This is particularly problematic when screening a large number of transformants [4]. Boiling algae with a buffer has the potential to disrupt the cell wall, allowing for the release of genomic DNA and thereby facilitating colony PCR detection. Hence, the practice of boiling microalgal cells has been commonly employed to aid in the liberation of genomic DNA, enabling its subsequent PCR amplification [4,5,6]. Nonetheless, research has shown that the effectiveness of colony PCR in microalgal cells after boiling can vary depending on their growth conditions. Notably, cells thriving under favorable growth conditions, such as exponential growth and early stationary phases, tend to yield more amplification products compared to those in less favorable conditions, like during stationary phase and under nitrogen deprivation [5].

In contrast, we have recently developed a novel approach known as Squash-PCR for microbial spore/colony PCR [7]. This method is built upon the “squash” technique, where whole cells are flattened on a glass surface and examined under a microscope. The effectiveness of Squash-PCR has been demonstrated across distinct filamentous fungal and yeast strains, and has consistently yielded high efficiency [7].

**Figure 1:**
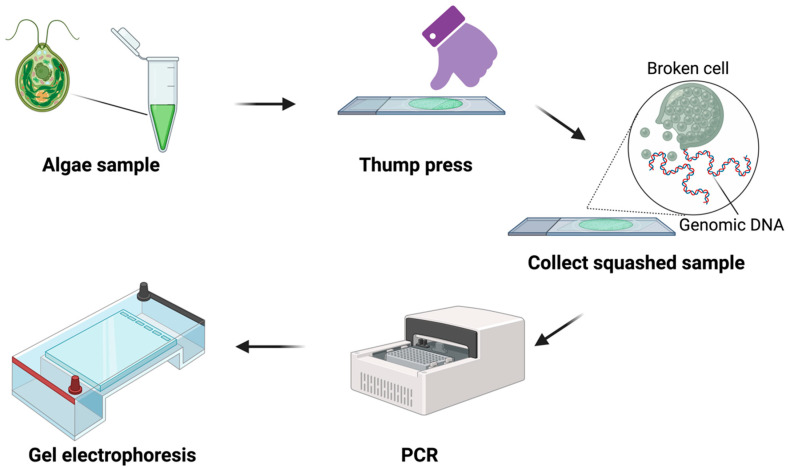
The workflow of Squash-PCR in microalgae.

In the current study, our primary objective is to expand the utility of the Squash-PCR method to the realm of microalgae. To facilitate a clear understanding of the typical procedures involved in squash preparation for Squash-PCR, we have provided a detailed illustration in Figure 1. In general, the pretreatment of microalgae takes approximately 1 min per sample. We believe that applying the Squash-PCR technique to microalgae will significantly improve our ability to screen for mutants and assess their genotypes efficiently.

## 2. Materials and Methods

### 2.1. Strains

The microalgae strains used for Squash-PCR were ordered from UTEX Culture Collection of Algae at UT-Austin and have been listed in Table 1.

### 2.2. Squash Preparation

Squash preparations were performed as follows: (1) Dispense ~ 4–5 μL of the microalgae cell solution onto a glass slide and cover it with a coverslip. (2) Apply firm, horizontal pressure to the coverslip using your thumb. (3) Remove the coverslip and add 10 μL of ultrapure water or PBS–Tween 20 solution (*v*/*v*, 0.05%). (4) Collect the cell solution with a pipette and dilute it with 10 μL of ultrapure water or PBS–Tween 20 as required. For more detailed instructions, refer to Yuan et al. [7].

### 2.3. Genomic DNA Extraction

A rapid plant DNA extraction method was employed to extract genomic DNA from all tested microalgal strains [8]. The protocol was as follows: collect a 200–500 μL microalgae sample in a 1.5 mL centrifuge tube and subject it to centrifugation at maximum speed (12,000–16,000× *g*). Discard the supernatant and proceed by grinding the sample using liquid nitrogen and a pestle. Subsequently, add 400 μL of the extraction buffer to the ground sample and incubate it at 60 °C for 15 min. The extraction buffer consists of a composition of 200 mM Tris-HCl, 200 mM NaCl, 25 mM EDTA, and 0.5% SDS (sodium dodecyl sulfate). Centrifuge the mixture at 4000 rpm to pellet the tissue. Transfer 300 μL of the supernatant to a new 1.5 mL centrifuge tube and add an equal volume of isopropanol. Recap the tubes and gently invert them several times to ensure thorough mixing. Allow the mixture to incubate at room temperature for 5 min. Following this, centrifuge the sample at 4000 rpm to pellet the DNA. Carefully pour off the supernatant without disturbing the pelleted DNA. Next, add 500 μL of 70% ethanol to each sample. Cover the tubes with clean caps and gently invert them to facilitate washing of the DNA. Centrifuge the samples at 4000 rpm for a washing cycle. Place the tubes flat on a paper towel and cover the open tubes with a KimWipe. Once the pellet is dry and no residual liquid remains in the tubes, proceed to re-suspend the pellets using 50 µL of TE buffer or nanopure H_2_O.

### 2.4. PCR Procedure

Water is utilized as a negative control during PCR. Genomic DNA extracted using SDS-method (as described in Section 2.3) was used as a positive control for each algae strain. Direct-PCR utilizes the algae solution (1 μL/reaction, ranging from 1 × 10^5^ to 1 × 10^7^ cells/mL) as the DNA template for PCR without any pre-treatment prior to the reaction. The DNA template of Squash-PCR was prepared using squash preparation described in Section 2.2. Dilution is applied for some of the tested samples after squashing.

For the PCR reactions in this study, we used GoTaq Green Master Mix from Promega. The PCR cycling conditions were as follows: an initial denaturation at 95 °C for 2 min, followed by 35 cycles of denaturation at 95 °C for 30 s, annealing at 55 °C for 30 s, and extension at 72 °C for 1 min, with a final elongation step at 72 °C for 5 min. Each reaction employed 1 μL of cell solution in a 20 μL reaction.

### 2.5. Primers for PCR

All primers used for microalgal Squash-PCR are listed in Table 2. The primers have been manually designed utilizing SnapGene software (https://www.snapgene.com/) and had a melting temperature (Tm) range between 55 and 60 °C.

### 2.6. Microscopy

Microscopic examination was employed to evaluate the condition of the microalgal cells, both prior to and following the squashing procedure. An Olympus BX60 microscope was utilized to examine samples and collect images. Initially, a volume of 5 to 10 μL of the microalgae sample was pipetted onto a microscope slide, followed by the placement of a coverslip. The slide was examined under a 40X objective lens to capture the initial cellular state. Subsequently, the sample underwent a squashing process by firmly pressing the coverslip using a thumb. This action was performed to disrupt the cell wall, facilitating the release of genomic DNA into the solution. The slide was once again examined under the same 40X objective lens setting to observe and document any discernible alterations in cellular morphology resulting from the squashing procedure.

### 2.7. Cell Counting

Cell counts were determined using a hemocytometer and a microscope. Cell concentrations were determined as previously described [9]. Briefly, the procedure was as follows: Start by vortexing the target cell suspension to ensure homogeneity. Next, pipette 5 μL of the cell sample into 995 μL of water and thoroughly mix it by vortexing. Place a glass coverslip over the counting chambers of a hemocytometer. Following this, pipette 10 μL of the cell sample into the hemocytometer and allow 30 s for the cells to settle. Subsequently, position the hemocytometer under a microscope using a 40X objective lens. Manually count the cells in the sample. Note that if cells touch the perimeter sides of a corner square, count cells only on two sides, either the two outer sides or the two inner sides, to ensure accuracy. To calculate cell concentration (cells/mL), multiply the total number of cells by the dilution factor and then by 10^4^. Finally, clean the hemocytometer and glass coverslip using 70% ethanol to ensure proper sterilization and preparation for subsequent use.

## 3. Results

### 3.1. DNA Template Preparation via Squash Technique

**Figure 2:**
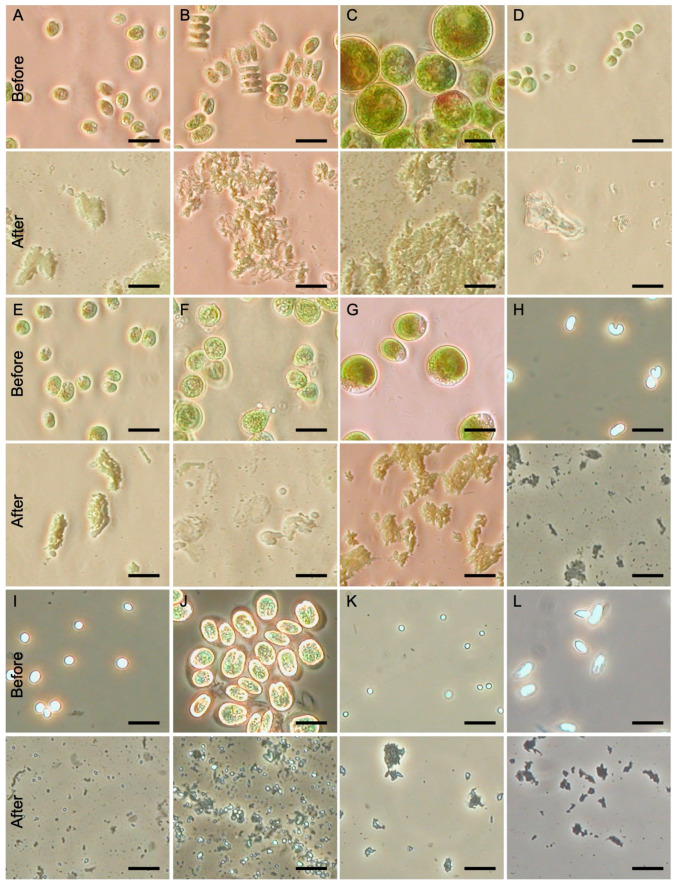
Microscopic observation of various microalgae strains in squash preparations. (**A**) *Chlamydomonas reinhardtii* UTEX 89. (**B**) *Scenedesmus* sp. UTEX 1589. (**C**) *Haematococcus pluvialis* UTEX 2505. (**D**) *Chlorella vulgaris* UTEX 395. (**E**) *Chlamydomonas reinhardtii* UTEX 90. (**F**) *Botryococcus braunii* UTEX 572. (**G**) *Dunaliella salina* UTEX LB 200. (**H**) *Monoraphidium minutum* 26B-AM. (**I**) *Chlorella sorokiniana* DOE1412. (**J**) *Tetraselmis striata* LANL1001. (**K**) *Picochlorum celeri* TG2. (**L**) *Scenedesmus obliquus* UTEX 393. Scale bar = 20 µm.

To evaluate the versatility and applicability of the Squash-PCR method across various microalgae species, we carefully selected a diverse set of 12 microalgae strains for our testing (Table 1). Each strain was chosen due to its importance to or potential utility in various industrial applications. For colony PCR to be effective, it is crucial that the genomic DNA from microalgae cells is thoroughly released and extracted before advancing to the PCR amplification step. This initial DNA extraction is a pivotal prerequisite, as the effectiveness of DNA release significantly influences the accuracy and reliability of downstream genetic analyses and manipulations. Following the procedures described in Figure 1, we subjected 12 distinct microalgae strains to the squashing process. To assess the efficacy of the squash preparation, we conducted a comparative analysis of cell samples both before and after squashing under a microscope (Figure 2). The microalgae strains we examined exhibited characteristics typical of single-celled, green microalgae and often presented a spherical or ellipsoidal shape before squashing (Figure 2). Notably, these cells spanned a diverse size and shape, with diameters ranging from 2 to 20 µm. Furthermore, this size variability was not confined to distinct populations but could even be observed within a single group of microalgae. This considerable diversity underscores the importance of employing a versatile and effective technique like squash technique, which can be applied to microalgae of varying shapes and sizes, enhancing its utility in genetic research across different species. Following the squashing process, all tested algae samples exhibited complete cell disruption, with no intact cells remaining observable under the microscope (Figure 2). This outcome indicates the effectiveness of the squash technique in achieving comprehensive cell disruption, a critical step in genetic research and colony PCR.

### 3.2. Efficacy of Squash-PCR in Diverse Microalgae Strains

**Figure 3:**
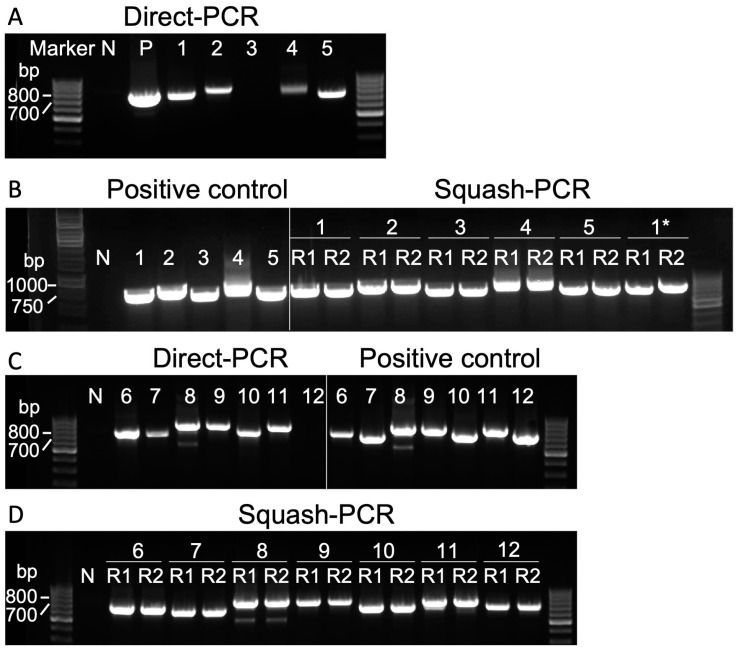
Squash-PCR of different microalgae strains using primers targeting ITS2 region. (**A**) Direct PCR using the cell suspensions of strain 1 to 5 without any treatment. P, the genomic DNA of strain 1 was extracted using SDS, serving as a positive control. (**B**) Squash-PCR of strains 1 to 5. (**C**) Direct PCR using the cell suspensions of strains 6 to 12 without any treatment. (**D**) Squash-PCR of strains 6 to 12. N, negative control; *, the samples underwent a 5-fold dilution following the squashing process; Squash-PCR was performed with two replicates, labeled as R1 and R2.

To assess the effectiveness of Squash-PCR, we employed a specific primer pair (102_F and 103_R) designed for the amplification of the internal transcribed spacer 2 (ITS2) region [10]. The ITS2 region, which is a highly conserved segment of ribosomal DNA but exhibits variability between different species, serves as a valuable genetic marker for distinguishing and identifying various microorganisms, including microalgae [11]. To rigorously evaluate Squash-PCR’s effectiveness, genomic DNA was initially extracted from all tested strains using a widely employed SDS-based DNA extraction method, serving as a positive control in our study. In the direct PCR of strains 1 to 5, strains 1 and 5 produced sharp PCR bands, while strains 2 and 4 showed relatively weaker bands, and strain 3 did not show any band (Figure 3A). In contrast, all five tested strains displayed sharp bands, comparable to the positive control samples, which were visibly more distinct than the results obtained through direct PCR (Figure 3A). In a similar manner, we conducted direct PCR for strains 6 to 12. Among these strains, strain 6, 8, 9, 10, and 11 exhibited sharp PCR bands, indicating successful amplification (Figure 3B). However, we observed relatively weak bands for strain 7 and no bands for strain 12 in the direct PCR reactions. In contrast, employing the Squash-PCR method for strains 6 to 12 resulted in consistently sharp bands, equivalent to those of the positive control samples (Figure 3B). In line with the findings observed in strains 1 to 5, the results obtained through Squash-PCR for strains 6 to 12 exhibited clearer and more distinct outcomes compared to the direct PCR approach. Overall, Squash-PCR consistently outperformed direct PCR in producing clear and distinct bands across all tested strains.

### 3.3. Evaluation of Squash-PCR in Microalgae Using Different Cell Numbers and Primer Pairs

**Figure 4:**
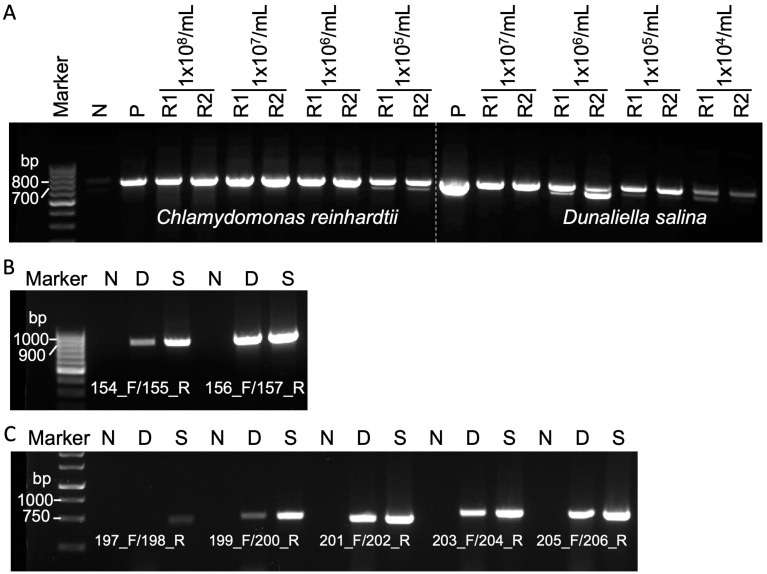
Investigation of potential limiting factors affecting Squash-PCR in microalgae. (**A**) The effective range of cell concentration for Squash-PCR in *Chlamydomonas reinhardtii* and *Dunaliella salina*. Squash-PCR was performed with two replicates, labeled as R1 and R2. (**B**) The examination of Squash-PCR in *Chlamydomonas reinhardtii* with different primers. (**C**) The examination of Squash-PCR in *Dunaliella salina* with different primers. N, negative control; P, positive control; D, direct PCR; S, Squash-PCR.

The performance of Squash-PCR may be influenced by factors such as cell number and the specific primer pairs used. In this study, we evaluated the Squash-PCR technique (with primers 102_F and 103_R) using different cell concentrations for two microalgae species, *Chlamydomonas reinhardtii* (ranging from 1 × 10^5^ to 10^8^ cells/mL) and *Dunaliella salina* (ranging from 1 × 10^4^ to 10^7^ cells/mL). In the case of *Chlamydomonas reinhardtii*, we observed sharp bands across all tested concentrations. Particularly, the concentration of 1 × 10^7^ cells/mL exhibited the highest abundance of PCR products, followed by 1 × 10^8^, 1 × 10^6^, and 1 × 10^5^ cells/mL, in decreasing order (Figure 4A). In *Dunaliella salina*, the intensity of PCR bands gradually decreased from the 1 × 10^7^ sample to the 1 × 10^4^ sample, with only very faint bands observed at 1 × 10^4^ cells/mL (Figure 4A). Taken together, effective PCR amplification is expected to be achieved when working with cell concentrations ranging from 1 × 10^5^ to 10^8^ cells/mL.

Furthermore, we conducted experiments with different primer pairs in both *Chlamydomonas reinhardtii* and *Dunaliella salina*. In *Chlamydomonas reinhardtii*, sharp bands were obtained using primers 154_F/155_R in the Squash-PCR method, while only a faint band was detected in direct PCR. However, both direct PCR and Squash-PCR using primers 156_F/157_R resulted in sharp bands (Figure 4B). Subsequently, five different primer pairs (197_F/198_R, 199_F/200_R, 201_F/202_R, 203_F/204_R, and 205_F/206_R) were employed to test Squash-PCR in *Dunaliella salina* (Figure 4C). Sharp PCR bands were observed in both direct PCR and Squash-PCR when using primer pairs 201_F/202_R, 203_F/204_R, and 205_F/206_R. In contrast, sharp bands were exclusively seen in Squash-PCR but not in direct PCR with primer pair 199_F/200_R. However, no sharp bands were detected in either direct PCR or Squash-PCR when employing primers 197_F/198_R. Notably, Squash-PCR consistently yielded higher amounts of PCR products compared to direct PCR. The increased PCR product yield in Squash-PCR further emphasizes the technique’s utility.

## 4. Discussion

PCR is a fundamental technique in microbiology and molecular biology with applications spanning genetic transformation validation, strain profiling, mutation screening, microalgal isolate selection, and quantitative analyses. PCR is a firmly established tool used to advance microalgae research [4,12,13,14,15,16,17]. Therefore, developing a more streamlined, efficient, and robust PCR method is vital for advancing and accelerating microalgae research. Having an adequate amount of microalgae DNA as the requisite template is the first step for successful PCR amplification. Various cetyltrimethylammonium bromide (CTAB)/SDS-based methods have been frequently reported and modified to extract microalgae DNA [18,19]. Although these methods can yield large quantities of high-quality genomic DNA, they often require several days for extended cell culturing steps and the need for whole-cell lysis methods, including mechanical homogenization, organic solvents, enzymatic digestion, or a combination of these methods [18].

Colony PCR is a well-established method primarily utilized for the rapid screening of genetically transformed colonies that is more commonly applied in bacteria or yeast research. Unlike typical colony PCR used with bacteria or yeast, the process used with microalgae colonies usually involves additional treatments before PCR can be performed. Multiple colony PCR methods have been specifically adapted for some microalgae. These methods generally involve the resuspension of cells using different lysis buffers, such as 10 mM Ethylenediaminetetraacetic acid (EDTA), 5–6% Chelex-100, Tris/EDTA (TE), 0.2 % SDS, 0.2% Triton X-100 or yeast protein extraction buffer (Y-PER), followed by a critical boiling step to promote cell lysis [14,20,21]. Among the various lysis buffers assessed, Y-PER has demonstrated superior effectiveness compared to other methods [21]. Furthermore, the boiling time has been proven to be a critical factor for successful colony PCR. The optimal boiling time for *Haematococcus pluvialis*, *Nannochloropsis salina*, and *Pseudochlorococcum* sp. is 10 min, whereas for *Nannochloropsis oculata*, 20 min is needed [5]. It is worth noting that algal cells cultivated under unfavorable conditions often develop a thicker secondary cell wall, which can potentially reduce the efficiency of genomic DNA release through boiling and subsequent PCR amplification [5,22]. Therefore, the selection of an appropriate lysis buffer and the optimal boiling time must be based on the characteristics of the specific microalgal strain under investigation.

In contrast, the squash preparation method detailed in this study has proven its versatility in effectively disrupting microalgal cells and enabling the release of genomic DNA. The utilization of Squash-PCR in microalgae strains presents several noteworthy benefits and advantages when compared to previously reported methods. This technique showcases its utility across a diverse array of microalgae species, regardless of variations in cell size, shape, or the complexity of the cell wall. Microalgal cells, with sizes ranging from as small as 2 μm to as large as 20 μm, can be effectively disrupted through squash preparation (Figure 2). Compared to traditional DNA extraction methods that typically necessitate a certain amount of biomass, often starting from a colony [18], Squash-PCR offers a substantial reduction in both labor and turnaround time, transforming the process from several days to just a matter of minutes (approximately 1 min per sample). This time-saving feature makes Squash-PCR a compelling choice for expediting microalgal research and streamlining genetic analysis. In contrast to colony PCR methods that rely on lysis buffers and boiling steps, Squash-PCR eliminates the influence of various lysis buffers, boiling times, and the choice of microalgal species on PCR amplification. This simplification of the process enhances the reliability and versatility of Squash-PCR in microalgal research. The present format of Squash-PCR is highly effective for processing a small number of samples. Recognizing the growing demand for screening large numbers of samples in the field of metabolic engineering, our forthcoming endeavor focuses on adapting this robust method for high-throughput screening. This adaptation will be achieved with the collaboration of mechanical engineers, who will contribute their expertise to enhance the method’s scalability and efficiency.

Interestingly, direct PCR using untreated microalgae cells yielded promising PCR amplification in some of the tested strains such as *Monoraphidium minutum* 26B-AM, *Scenedesmus obliquus* UTEX 393, *Chlorella vulgaris* UTEX 395, *Chlamydomonas reinhardtii* UTEX 90, and *Botryococcus braunii* UTEX 572 (Table 1). This outcome differs from previous reports where none of the tested strains displayed any PCR bands when samples were not boiled [5]. This variation may be attributed to the utilization of distinct strains, variations in culture conditions, lysed cells due to stress development, and differences in PCR enzymes and buffers. Regardless of whether direct PCR was successful, Squash-PCR consistently showed a significant increase in PCR amplification in all tested strains. This enhanced sensitivity ensures reliable mutant screening. Squash-PCR is particularly useful when working with specific microalgae strains in which obtaining PCR-quality DNA samples can be challenging due to their complex cell wall structures.

Notably, the squash preparation is effective for releasing and extracting PCR-quality genomic DNA. Nevertheless, it may not be the best choice when large quantities of high-quality DNA are required, such as for Next-Generation Sequencing (NGS) applications.

## 5. Conclusions

In this study, we introduce Squash-PCR, an innovative method that enhances microalgal research by simplifying DNA extraction and improving PCR efficiency. It has versatility and applicability to various microalgal species and simplifies strain engineering by providing an efficient and reliable genomic DNA release method. With improved PCR product detection and adaptability to different conditions, Squash-PCR is valuable for industrial applications like biofuels, pharmaceuticals, and biotechnology. Overall, this adaptability makes it a versatile technique for a broad range of microalgal strains in both industry and research.

## Figures and Tables

**Table 1:** Results of Squash-PCR in different microalgae strains.

No.	Microalgae Species	^1^ Direct-PCR	Squash-PCR
1	*Monoraphidium minutum* 26B-AM	+	++
2	*Chlorella sorokiniana* DOE1412	+ *	++
3	*Tetraselmis striata* LANL1001	−	++
4	*Picochlorum celeri* TG2	+ *	++
5	*Scenedesmus obliquus* UTEX 393	+	++
6	*Chlamydomonas reinhardtii* UTEX 89	+	++
7	*Scenedesmus* sp. UTEX 1589	+ *	++
8	*Haematococcus pluvialis* UTEX 2505	+	++
9	*Chlorella vulgaris* UTEX 395	+	++
10	*Chlamydomonas reinhardtii* UTEX 90	+	++
11	*Botryococcus braunii* UTEX 572	+	++
12	*Dunaliella salina* UTEX LB 200	−	++

+: Indicates a positive PCR band. −: Indicates a negative PCR band. ++: Represents a brighter PCR band. *: Indicates a faint PCR band. ^1^ Direct-PCR: PCR utilizing the algae solution directly as the DNA template without any pre-treatment before the PCR reaction.

**Table 2:** The oligos used for PCR in this study.

Primer	Sequence	Microalgae Species	Gene	Size (bp)
102_F	AGGAGAAGTCGTAACAAGGT	Strains 1 to 12	*ITS2*	700–900
103_R	TCCTCCGCTTATTGATATGC			
154_F	TTGCACACAAGAACGCATGA	*Chlamydomonas* *reinhardtii*	*FTSY*	815
155_R	GGCATTGGAGTAAACGACCC			
156_F	CTACGTCGCCTTACTGTGTG	*Chlamydomonas* *reinhardtii*	*ZEP*	809
157_R	GATTGCCTACTCACCACTCG			
197_F	ATGGTTCCACAAACTGAAACG	*Dunaliella salina*	*rbcL*	480
198_R	GTCACGCTCAACTTGAATACC			
199_F	CGTAGACTGTGTAGAAGCTG	*Dunaliella salina*	*orf121*	518
200_R	GTGGAACTACACCTTCAGGT			
201_F	ATGCCAATTGGTGTTCCAC	*Dunaliella salina*	*clpP*	496
202_R	GTGCATTAAAATCGTAAGGCG			
203_F	CAATGCGCACACCAGAAG	*Dunaliella salina*	*atpA*	551
204_R	GCATATAGTTTCCTGCGTCC			
205_F	ATGGCACGTGCTAAATTTGA	*Dunaliella salina*	*tufA*	524
206_R	GCAGAACCTGAAACGATAGG			

## Data Availability

Data are contained within the article.
